# Draft Genome Sequence of Plesiomonas shigelloides Strain PI-19, Isolated from the Intestine of Pufferfish (Tetraodon cutcutia)

**DOI:** 10.1128/mra.01252-21

**Published:** 2022-03-02

**Authors:** Tanmoy Debnath, Subrata K. Das

**Affiliations:** a Institute of Life Sciences, Department of Biotechnology, Bhubaneswar, India; b Regional Center for Biotechnology, NCR Biotech Science Cluster, Faridabad, Haryana, India; University of Arizona

## Abstract

We report the draft genome sequence of Plesiomonas shigelloides strain PI-19, which was isolated from the intestine of freshwater pufferfish. The genome of strain PI-19 contain 3,146 protein-coding sequences and harbors virulence genes associated with pathogenicity.

## ANNOUNCEMENT

The genus Plesiomonas is represented by a single species in the family *Enterobacteriaceae*. Plesiomonas shigelloides is a Gram-negative, facultative anaerobic, rod-shaped bacterium. It was identified as a potential human and animal pathogen (1). The isolation of Plesiomonas shigelloides from cultured fishes (Ctenopharyngodo nidellus and Orechromis nilotica) during mass mortality events indicates that this bacterium is highly pathogenic (2). *Plesiomonas* sp. strain PI-19 was isolated from the intestine of a freshwater pufferfish. For the isolation of bacteria, pufferfish were sacrificed and dissected aseptically, and the intestinal tissues were suspended in 2 mL sterile phosphate-buffered saline (PBS). The tissues were homogenized and used as a stock solution to isolate bacteria. An aliquot (100 μL) of gut tissue homogenate was serially diluted using PBS and plated onto nutrient agar (BD, Difco) to isolate bacteria. All plates were incubated at 28°C (corresponding to the river water temperature) for 2 days. Morphologically unique single colonies that appeared on nutrient agar plates were picked up, purified, and preserved in 15% glycerol at −80°C (3). *Plesiomonas* sp. strain PI-19 was grown overnight in 100 mL nutrient broth (BD, Difco). The cell suspension was then centrifuged at 8,000 rpm, and the culture pellet was used to isolate DNA following the methods of Sambrook and Russel (4). The DNA concentration and quality were measured using a NanoDrop 8000 spectrophotometer (Thermo Fisher Scientific). A combination of short-read Illumina and long-read Oxford Nanopore Technologies sequencing platforms was used to generate the complete genome sequence of *Plesiomonas* sp. strain PI-19. Illumina short-read DNA sequencing was carried out using the Illumina HiSeq 4000 next-generation sequencing platform to produce paired-end sequence reads with 150-bp read length. Read quality was assessed with the FastQC v0.11.5 program (http://www.bioinformatics.babraham.ac.uk/projects/fastqc) using default parameters. Low-quality bases and adapters were removed with Trimmomatic v0.36 (5) using the parameters mentioned in the manual. Trimmed reads were assembled *de novo* using Unicycler v0.4.7 (6), followed by assembly polishing with Pilon v1.23 (7). Assembly quality was assessed with QUAST v4.6.1 (8). For long-read Nanopore sequencing, a genomic library was prepared using the Nanopore ligation sequencing kit (SQK-LSK109; Oxford Nanopore Technologies, Oxford, UK). The library was sequenced with an R9.4.1 MinION flow cell (FLO-MIN106) using MinKNOW v2.0 with default settings. Barcode and adapter sequences were trimmed from Nanopore long reads using Porechop v0.2 (https://github.com/rrwick/Porechop), and reads with a minimum length of 1 kb were filtered using seqtk v1.2 (https://github.com/lh3/seqtk) for downstream analysis. The raw read *N*_50_ value was 718 bp. The hybrid genome assembly was performed using Unicycler v0.4.9 in hybrid assembly mode (6). The highly accurate Illumina short reads were aligned against the long Nanopore reads to sort out random sequencing errors (6). The assembled genomes were annotated using the NCBI Prokaryotic Genome Annotation Pipeline (PGAP) v4.9 with default parameters (9). The completeness and contamination of the whole-genome sequence were measured using CheckM (10). The genomic G+C content and assembly statistics were determined using a Perl script (https://github.com/tomdeman-bio/Sequence-scripts/blob/master/calc_N50_GC_genomesize.pl). A total of 2,016,165 Illumina reads and 1,378,263 Nanopore reads were assembled, which generated 8 scaffolds with an *N*_50_ value of 3,384,982 bp. The genome is 3.78 Mb, with a G+C content of 51.72%. The final draft genome contains 3,146 protein-coding sequences, 119 tRNAs, and the following rRNAs: 5S rRNAs, 12; 16S rRNAs, 12; 23S rRNAs, 13.

Comparative genomic analysis was performed with the genome sequences of type strains available in the NCBI database, to evaluate the genomic relatedness, using JSpeciesWS (11). The average nucleotide identities between *Plesiomonas* sp. strain PI-19 and the reference genomes were >96%, indicating the same species as Plesiomonas shigelloides 14029^T^ (12). Further, *in silico* DNA-DNA hybridization (DDH) was performed with the reference strains to calculate the genome-to-genome distances (13). *In silico* DDH values were >70%, confirming the same species as Plesiomonas shigelloides 14029^T^. Strain PI-19 also designated TBRC 15627 or NBRC 115343.

This study was carried out with approval from the institutional animal ethics committee (letter V-311-MISC/2017-18/ILS/884).

### Data availability.

The whole-genome shotgun project for *Plesiomonas* sp. strain PI-19 has been deposited in DDBJ/ENA/GenBank under the accession number JAJNMQ000000000. The version described in this paper is version JAJNMQ000000000.1. Illumina and Nanopore sequencing data are available in the NCBI SRA database under the accession numbers SRR17381640 and SRR17381641, respectively.
